# Accelerating target discovery using pre-competitive open science—patients need faster innovation more than anyone else

**DOI:** 10.3332/ecancer.2016.ed57

**Published:** 2016-08-03

**Authors:** Eric Low, Chas Bountra, Wen Hwa Lee

**Affiliations:** 1 Myeloma UK, 22 Logie Mill, Edinburgh EH7 4HG, UK.; 2 Structural Genomics Consortium, University of Oxford, Old Road Campus, Oxford OX3 7DQ, UK.

## Abstract

We are experiencing a new era enabled by unencumbered access to high quality data through the emergence of open science initiatives in the historically challenging area of early stage drug discovery. At the same time, many patient-centric organisations are taking matters into their own hands by participating in, enabling and funding research. Here we present the rationale behind the innovative partnership between the Structural Genomics Consortium (SGC)—an open, pre-competitive pre-clinical research consortium and the research-focused patient organisation Myeloma UK to create a new, comprehensive platform to accelerate the discovery and development of new treatments for multiple myeloma.

For anyone involved in biomedicine, especially in oncology, it seems like every other day we hear reports in the media about a new wonder drug or a potential cure. What is more, when scientists and clinicians attend conferences and read research journals, there is a seemingly endless amount of data being presented on the results of research, but still progress towards finding cures seems painfully slow. Why is this? One reason could be that the research or trials being done are on the same tired handful of targets and mechanisms.

Of course, on every scientist and clinician’s quest to develop faster, better treatments, the most common approach is to tread a familiar route and work on previously validated targets and mechanisms [1]. However in recent years, this has evolved to be almost the only path followed by scientists, academic and industrial alike. This is mainly as a consequence of the constantly increasing pressure from investors and funders (public and private) to deliver ‘return on investment’ as quickly as possible [2].

This has resulted in the competitive line being pushed too far back into the early stages of biomedical research and drug discovery meaning that groups of researchers are now working and thinking in isolation and in secrecy rather than working together and aggregating resources and brain power to better understand and unlock the incredibly complex human biology in a timeframe that means more to patients [3, 4].

However, recently, “comeback” movements, mostly born out of the lack of success of the old slower and more cumbersome model, have been gaining very strong traction. These new models look very different and increasingly rely on several organisations working in partnerships under so called ‘Open Innovation’ frameworks, varying from resource-sharing collaborations to plain and unambiguous pre-competitive agreements in the absence of patents [5, 6].

One of the most unrestricted initiatives is the Structural Genomics Consortium (SGC), which has been focusing on exploring areas of biology previously under-explored for drug development since 2004. The SGC has been generating high quality, validated and reproducible molecular ‘toolkits’ for novel genes/proteins [7], including human recombinant proteins, crystallographic structures of such proteins, functional assays, small molecule binders (e.g. inhibitors), and renewable antibodies. Each toolkit revolving around a new protein is then called a ‘Target Enabling Package—TEP’.

The TEPs and all the outputs generated as a result of the exploitation of these toolkits by research collaborations and partnerships are made publicly available immediately, without any patents or restrictions, alongside the protocols to reproduce them. This completely transparent strategy has already enable crowdsourced exploration of completely novel targets, in a disease-agnostic manner.

In one of the fastest developments in uncovering novel therapeutic protein target families, the pioneer BRD4 inhibitor JQ1 was developed by the SGC and collaborators, which was quickly followed by a steady stream of many other novel, fully-open inhibitors for several other members of the bromodomain family [8, 9] (i.e. fully disclosing chemical structure, physical and chemical properties, selectivity profile, cell-based assay data, biological characterisation, etc.).

To date, the SGC and its industrial and academic partners have already placed more than 40 novel epigenetics probes in the public domain (http://www.thesgc.org/chemical-probes) and are working to further expand this set beyond epigenetic targets.

This science-first, open approach has brought together many of the world’s best industrial and academic scientists to decipher the biology, thus creating more innovative starting points to accelerate translation.

In the meantime, another type of grassroots movement has evolved in the form of disease-specific, patient–driven organisations, increasingly frustrated at the lack of progress and success of the current drug discovery and development model.

These efforts have stemmed from patient support groups who have developed into fully-fledged research-oriented organisations and who have moved beyond the historical reactive grant-giving model to organisations that are driving, leading and funding cutting-edge, patient-centric disease- specific research agendas.

They have understood how to leverage academia and health systems to do cost-effective research and to build meaningful and professional collaborations and partnerships with industry. They have also harnessed the broad concepts and principles of venture capitalism to form a not-for-profit version of ‘venture philanthropy’ which now gives patient-driven research organisations the ability to be research investors and not just funders.

What is more, these new models have given patients a seat at the table and they are fast becoming the broker with the ability and the nous to align stakeholder interests, incentives and rewards around a common goal—patients.

One notable example of this is Myeloma UK. Since its inception, Myeloma UK has adopted a full-on approach to recreating a complete drug discovery and development infrastructure, firstly building on and strengthening their own clinical research capabilities and extending the network of myeloma clinical experts, then moving to reinforce the generation of new clinical candidates based on existing targets and combination approaches.

However, Myeloma UK realised that the chokepoint in myeloma was the limited number of novel targets being prosecuted and as a result, decided to implement the final piece in its strategy—an early-stage target discovery component.

This is when the SGC and Myeloma UK first discussed the potential to partner by bringing together the matured and sophisticated research capability and platforms from either side to multiply their impacts (Figure 1): the SGC’s disease agnostic approach with all the novel targets and technologies developed in its extensive research platform implemented in six leading academic institutions across the globe with the disease-specific knowledge, research infrastructure and tools as well as the drive, passion and sense of urgency, in the Myeloma UK research model.

In the partnership, Myeloma UK is funding discovery scientists within the SGC network to carry out myeloma-focused research using the SGC platform and reagents, e.g. assessing the importance of epigenetics regulators as potential targets to treat myeloma or to enhance susceptibility of myeloma cells to existing treatments.

The Myeloma UK scientists at the SGC will also become ‘Knowledge Transferors’ themselves, working and managing the interface between the SGC’s global pre-clinical open science network (academic and industrial) and Myeloma UK’s own translational platforms and early phase trial capabilities. This will result in the creation of a new breed of Super-Translational scientists, straddling both networks, becoming open science champions for myeloma to attract and channel even more scientists worldwide to work for the cause.

The gift of Open Science is one that keeps giving, as other disease foundations join the network, we will experience a high acceleration in the creation of an open pool of research tools and disease-specific assays contributed by each foundations’ own network. With novel disease foundation partners, there will also be an increasing number of novel targets for which the SGC can generate a TEP, so further expanding the toolbox that can be shared by any of the disease foundations and research groups.

The SGC-Myeloma UK partnership is just part of the first chapter in the journey to create a new framework where previously passive stakeholders are in control of renewed and devolved responsibilities: in a Brave New World where free flow of knowledge has already enabled vertiginous progress in many areas, why should we conform to obsolete models of the past when drug discovery and patient benefits are at stake? It is time for a change and the authors hope the model presented above will inspire many others to join us in our quest to create new medicines for patients.

## Figures and Tables

**Figure 1:**
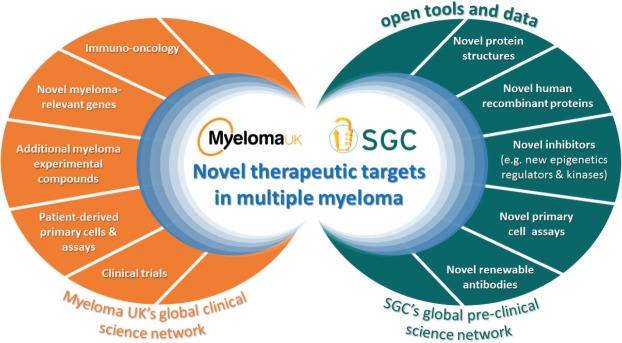
The Myeloma UK -SGC partnership: a new framework to accelerate discovery of novel therapeutic targets through openly sharing tools and data to leverage and expand the myeloma research community.
